# Sliding Mode Observer with Exponential Reaching Law for Speed Estimation of a Six-Phase Induction Machine

**DOI:** 10.3390/s26144513

**Published:** 2026-07-16

**Authors:** Larizza Delorme, Magno Ayala, Osvaldo Gonzalez, Jorge Rodas, Ariel Fleitas, Raúl Gregor, Jesus C. Hernandez

**Affiliations:** 1 Centro de Investigación en Tecnologías Hidroeléctricas y Energía Distribuida—CITHED, Department of Electronics and Mechatronics Engineering, Facultad de Ingeniería, Universidad Nacional de Asunción, Luque 110948, Paraguay; mayala@ing.una.py (M.A.); ogonzalez@ing.una.py (O.G.); jrodas@ing.una.py (J.R.); afleitas@fiuna.edu.py (A.F.); rgregor@ing.una.py (R.G.); 2 Department of Electrical Engineering, University of Jaen, Campus Lagunillas s/n, Edificio A3, 23071 Jaen, Spain

**Keywords:** electric motor drives, exponential reaching law, six-phase induction machine, sliding-mode control, speed sensorless

## Abstract

High-performance sensorless operation in multiphase electric drives requires speed estimation techniques capable of providing fast dynamic response, reduced oscillatory behavior, and low implementation complexity. In this context, a sliding-mode observer (SMO) based on an exponential reaching law (ERL) is proposed for rotor speed estimation in asymmetrical six-phase induction machines operating under indirect rotor field-oriented control. Unlike conventional SMO implementations, the proposed approach avoids auxiliary low-pass filtering (LPF) stages by employing an ERL-based adaptive gain mechanism, thereby preventing the phase delay and bandwidth reduction commonly associated with LPF-based observers. As a result, the proposed observer preserves fast transient dynamics, attenuates chattering near the sliding surface, and improves the smoothness of the estimated signals. The proposed technique is particularly suitable for multiphase drive applications, where sensorless operation reduces hardware complexity and improves system reliability by eliminating mechanical speed sensors and associated wiring. A Lyapunov-based stability analysis is presented to demonstrate the convergence properties of the observer and discuss the influence of the ERL parameters on the estimation dynamics. Simulation and experimental results obtained on a real-time test bench validate the digital implementation of the proposed SMO + ERL, demonstrating improved transient tracking, smoother estimated signals, stable low-speed operation, satisfactory speed reversal performance, and effective operation under loaded conditions.

## 1. Introduction

The growing adoption of multiphase electric machine drives in high-performance applications is primarily motivated by their improved power distribution capability, reduced torque ripple, additional control degrees of freedom, and inherent fault-tolerant features compared with conventional three-phase systems [1,2]. These characteristics are particularly attractive in applications where reliability, availability, and power scalability are critical requirements. Representative examples include electric traction, where the stator current can be distributed across a larger number of phases to reduce the per-phase current rating and improve post-fault operating capability; marine electric propulsion and aerospace electromechanical actuators, where continuity of service under fault conditions is essential; and wind energy conversion systems, small hydropower generation, and high-power industrial drives, where modular converter configurations and enhanced fault tolerance contribute to improved system availability [2,3,4,5,6,7]. In particular, asymmetrical six-phase induction machines (SPIMs) with isolated neutrals are especially suitable for medium- and high-power conversion systems because they can be supplied by two conventional three-phase voltage-source inverters (VSIs), enabling modular power conversion. Furthermore, the vector-space decomposition (VSD) framework enables independent regulation of the torque-producing and non-torque-producing subspaces, improving the flexibility of the control strategy [8,9].

Within this context, field-oriented control (FOC) remains one of the most widely adopted strategies for high-performance induction motor drives, while indirect rotor-flux-oriented control (IRFOC) is particularly attractive due to its relatively simple implementation and well-established industrial applicability [10]. Nevertheless, high-performance IRFOC requires accurate rotor speed information for both the outer speed regulation loop and the synchronous reference-frame generation. In this context, the use of mechanical speed sensors increases hardware complexity, cost, and maintenance requirements, and limits their applicability in harsh operating environments [11]. Consequently, sensorless control strategies based on state observers have become an active research topic for electric drives.

Accordingly, several observer-based sensorless strategies for multiphase machines have also been reported in the literature, including extended Kalman filter (EKF)-based estimation schemes, model reference adaptive systems (MRAS), adaptive rotor-parameter identification techniques, and fault-tolerant sensorless control approaches under phase-loss conditions, among others [12,13,14,15,16]. Concurrently, emerging finite position set phase-locked loop (FPS-PLL) techniques have been recently developed to improve position tracking performance by eliminating conventional PI synchronization loops, although their application is predominantly restricted to permanent magnet synchronous motor drives due to their straightforward flux-position coupling [17,18]. Although these various methods provide satisfactory estimation performance, they are often associated with higher parameter tuning effort or increased computational complexity; the latter is a prominent limitation in both EKF algorithms and predictive search-based methods like the FPS-PLL, which may require intensive iterative processing within each sampling period [19]. Therefore, there remains significant interest in developing sensorless estimation techniques capable of providing fast dynamic response and reduced oscillatory behavior while maintaining low implementation complexity in multiphase drives.

Among the different sensorless approaches, sliding-mode observers (SMOs) have been extensively investigated due to their favorable dynamic behavior, inherent robustness features, accurate state reconstruction capability and suitability for real-time implementation in electric drives [20,21,22,23]. Recent studies focused on multiphase drives have demonstrated the feasibility of SMO-based speed estimation within VSD-based control structures, highlighting the practical considerations associated with multiphase modeling and implementation [24,25,26,27,28,29]. In particular, these studies confirm the potential of SMO techniques for multiphase machine drives; however, the number of reported contributions remains limited in the case of asymmetrical SPIM. From an implementation perspective, conventional SMO usually reconstruct the equivalent control signal by applying a low-pass filter (LPF) to the discontinuous switching term. Although this approach attenuates the high-frequency components associated with the switching action, it introduces phase delay and reduces the effective observer bandwidth, thereby degrading the estimation performance during low-speed operation and fast dynamic transients [30,31].

To overcome these limitations, various formulations have been investigated for sliding-mode control and observer design of the SPIM [32,33]. In particular, exponential reaching laws (ERLs) provide an adaptive convergence mechanism in which the observer gain evolves according to the magnitude of the sliding variable. This behavior allows stronger correction during large estimation errors while naturally reducing the effective switching activity as the trajectory approaches the sliding surface. Consequently, the ERL formulation improves the smoothness of the estimated signals and mitigates chattering effects without requiring additional low-pass filtering stages [25].

Motivated by these considerations, this paper proposes a speed-sensorless control strategy for an asymmetrical SPIM using a sliding-mode observer enhanced with an exponential reaching law (SMO + ERL). The main contributions of this work are summarized as follows:

- An SMO + ERL formulation for rotor speed estimation in asymmetrical SPIMs operating under IRFOC, where the classical LPF stage is eliminated through an ERL-based adaptive gain mechanism;
- A Lyapunov-based stability analysis establishing global reaching and convergence of the current and rotor-flux estimation errors while analyzing the influence of the ERL parameters on the observer dynamics and chattering attenuation;
- A comprehensive simulation and experimental validation under transient and steady-state operating conditions, demonstrating improved dynamic response, smoother estimation behavior, and reduced oscillatory effects compared with the conventional LPF-based SMO implementation.

The organization of this paper is outlined as follows. Section 2 presents the mathematical formulation of the asymmetrical SPIM drive and its associated dynamic model in the stationary reference frame. Section 3 details the proposed speed-sensorless control approach that combines IRFOC with the SMO + ERL observer. In Section 4, a Lyapunov-based stability analysis of the proposed SMO + ERL estimation scheme is carried out. Section 5 reports both simulation and experimental results obtained from the laboratory test bench under steady-state and transient operating conditions, including speed reversal and load variation tests. Section 6 discusses the performance of the proposed strategy using quantitative indicators, including tracking accuracy and current regulation quality. Finally, Section 7 summarizes the main conclusions and contributions of this work.

## 2. Mathematical Model of the SPIM

**Figure 1:**
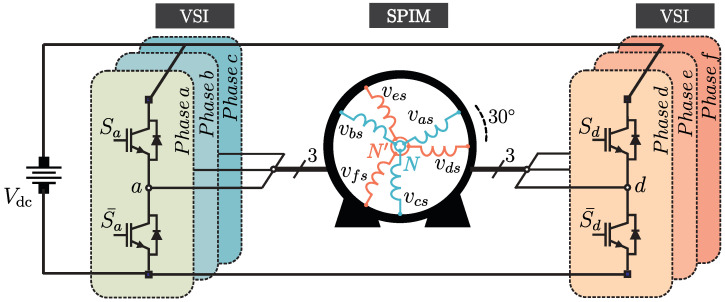
Configuration of the asymmetrical SPIM with a double three-phase two-level VSI.

The electric drive under study corresponds to an asymmetrical SPIM composed of two three-phase stator windings spatially displaced by 30° electrical with isolated neutral points, as shown in Figure 1. This configuration is widely adopted in multiphase drive applications due to its enhanced fault-tolerance capability, power-sharing characteristics, and reduced low-order harmonic content resulting from the spatial displacement between the two three-phase stator winding sets [34,35].

The dynamic behavior of the SPIM can be initially described in the phase domain by a set of differential equations with time-varying coefficients. Nevertheless, this representation is not suitable for control synthesis and observer design due to its high dimensionality and parameter coupling. To obtain a formulation with constant parameters, the vector space decomposition (VSD) approach is employed, thereby transforming the six-phase system into a set of orthogonal subspaces with clear, distinct physical interpretations. In this framework, the original phase variables are mapped into three independent two-dimensional subspaces [36]. The (α–β) subspace represents the fundamental energy conversion mechanism of the machine, as it is directly responsible for air-gap flux generation and electromagnetic torque production. Consequently, this subspace plays a dominant role in speed and torque control. The (x–y) subspace is associated with non-torque-producing harmonic components, which mainly contribute to additional copper losses and current distortion without participating in electromechanical energy conversion [37]. Finally, the $(z_{1}$–$z_{2})$ subspace corresponds to zero-sequence components; however, due to the isolated neutral configuration of the SPIM, these components do not circulate and therefore have no impact on the machine dynamics.

By applying an amplitude-invariant Clarke transformation to the stator and rotor phase variables, the electrical quantities are mapped into the (α–β), (x–y), and $(z_{1}$–$z_{2})$ subspaces. The transformation matrix C is defined as:

(1) $$C=\frac{1}{3}\left[\begin{array}{cccccc} 1 & \frac{\sqrt{3}}{2} & -\frac{1}{2} & -\frac{\sqrt{3}}{2} & -\frac{1}{2} & 0\\ 0 & \frac{1}{2} & \frac{\sqrt{3}}{2} & \frac{1}{2} & -\frac{\sqrt{3}}{2} & -1\\ 1 & -\frac{\sqrt{3}}{2} & -\frac{1}{2} & \frac{\sqrt{3}}{2} & -\frac{1}{2} & 0\\ 0 & \frac{1}{2} & -\frac{\sqrt{3}}{2} & \frac{1}{2} & \frac{\sqrt{3}}{2} & -1\\ 1 & 0 & 1 & 0 & 1 & 0\\ 0 & 1 & 0 & 1 & 0 & 1 \end{array}\right],$$

such that any electrical variable can be expressed as:

(2) $${\left[\begin{array}{cccccc} X_{\alpha } & X_{\beta } & X_{x} & X_{y} & X_{z1} & X_{z2} \end{array}\right]}^{T}=C{\left[\begin{array}{cccccc} X_{a} & X_{d} & X_{b} & X_{e} & X_{c} & X_{f} \end{array}\right]}^{T},$$

where X represents stator or rotor voltages, currents, or flux linkages.

Under these considerations, the stator voltage equations in the stationary reference frame can be written as:

(3) $$\begin{array}{cc} v_{\alpha s} & =R_{s}i_{\alpha s}+{\dot{\psi }}_{\alpha s}, \end{array}$$

(4) $$\begin{array}{cc} v_{\beta s} & =R_{s}i_{\beta s}+{\dot{\psi }}_{\beta s}, \end{array}$$

(5) $$\begin{array}{cc} v_{xs} & =R_{s}i_{xs}+{\dot{\psi }}_{xs}, \end{array}$$

(6) $$\begin{array}{cc} v_{ys} & =R_{s}i_{ys}+{\dot{\psi }}_{ys}, \end{array}$$

(7) $$\begin{array}{cc} v_{z1s} & =R_{s}i_{z1s}+{\dot{\psi }}_{z1s}, \end{array}$$

(8) $$\begin{array}{cc} v_{z2s} & =R_{s}i_{z2s}+{\dot{\psi }}_{z2s}. \end{array}$$

For the squirrel-cage rotor, the rotor voltages are zero, leading to the following equations in the (α–β) subspace:

(9) $$\begin{array}{cc} 0 & =R_{r}i_{\alpha r}+{\dot{\psi }}_{\alpha r}+\omega_{r}\psi_{\beta r}, \end{array}$$

(10) $$\begin{array}{cc} 0 & =R_{r}i_{\beta r}+{\dot{\psi }}_{\beta r}-\omega_{r}\psi_{\alpha r}, \end{array}$$

where $\omega_{r}$ denotes the electrical rotor speed. The flux linkages are related to the stator and rotor currents by:

(11) $$\begin{array}{cccc} \psi_{\alpha s} & =L_{s}i_{\alpha s}+Mi_{\alpha r},\;\;\;\;\;\;\;\;\;\;\;\;\;\;\;\;\;\; & \psi_{\beta s} & =L_{s}i_{\beta s}+Mi_{\beta r}, \end{array}$$

(12) $$\begin{array}{cccc} \psi_{\alpha r} & =L_{r}i_{\alpha r}+Mi_{\alpha s},\;\;\;\;\;\;\;\;\;\;\;\;\;\;\;\;\;\; & \psi_{\beta r} & =L_{r}i_{\beta r}+Mi_{\beta s}, \end{array}$$

(13) $$\begin{array}{cccc} \psi_{xs} & =L_{ls}i_{xs},\;\;\;\;\;\;\;\;\;\;\;\;\;\;\;\;\;\;\;\;\;\;\;\;\;\;\;\;\;\; & \psi_{ys} & =L_{ls}i_{ys},\;\;\;\;\;\;\;\;\;\;\;\; \end{array}$$

(14) $$\begin{array}{cccc} \psi_{z1s} & =L_{ls}i_{z1s},\;\;\;\;\;\;\;\;\;\;\;\;\;\;\;\;\;\;\;\;\;\;\;\;\;\;\; & \psi_{z2s} & =L_{ls}i_{z2s},\;\;\;\;\;\;\;\;\;\;\;\;\;\;\; \end{array}$$

where $M=3L_{m}$ is the mutual inductance, $L_{s}=L_{ls}+M$ and $L_{r}=L_{lr}+M$ are the stator and rotor inductances, respectively.

The electromagnetic torque developed by the SPIM depends exclusively on the (α–β) components and is expressed as:

(15) $$T_{em}=\frac{6}{2}PM\left(i_{\alpha r}i_{\beta s}-i_{\beta r}i_{\alpha s}\right),$$

where *P* is the number of pole pairs.

Finally, the mechanical dynamics of the machine are governed by:

(16) $$T_{em}-T_{L}=J{\dot{\omega }}_{m}+B\omega_{m},\;$$

with $T_{L}$ being the load torque, *J* the moment of inertia, and *B* the viscous friction coefficient. The mechanical and electrical rotor speeds are related by:

(17) $$\omega_{m}=\frac{\omega_{r}}{P},\;$$

which allows the mechanical equation to be rewritten directly in terms of the electrical speed as:

(18) $$T_{em}-T_{L}=\frac{J}{P}{\dot{\omega }}_{r}+\frac{B}{P}\omega_{r}.$$

## 3. Proposed Speed Sensorless Control Strategy

High-performance operation of multiphase electric drives requires a closed-loop control structure capable of accurately regulating torque and speed under a wide range of operating conditions. Conventionally, such control schemes rely on direct measurement of the rotor speed using mechanical sensors. When this information is replaced with an observer-estimated signal, the drive operates in a sensorless configuration while preserving the overall control architecture.

In the proposed approach, the control system is organized in a cascaded structure composed of an outer speed regulation loop and inner current control loops. The estimated rotor speed is used both for speed feedback and for reference frame synchronization. The voltage references generated by the control algorithm are applied to the six-phase VSI through a modulation stage. Although some advanced methods compute switching states directly, a carrier-based pulse width modulation (CB-PWM) strategy is adopted in this work for its simplicity and suitability for real-time digital implementation.

### 3.1. Indirect Rotor Field-Oriented Control (IRFOC)

IRFOC is employed as the baseline vector control strategy. This method, originally developed for three-phase induction machines, has been extensively adapted to multiphase drives and offers a convenient framework for decoupled control of electromagnetic torque and rotor flux [37].

**Figure 2:**
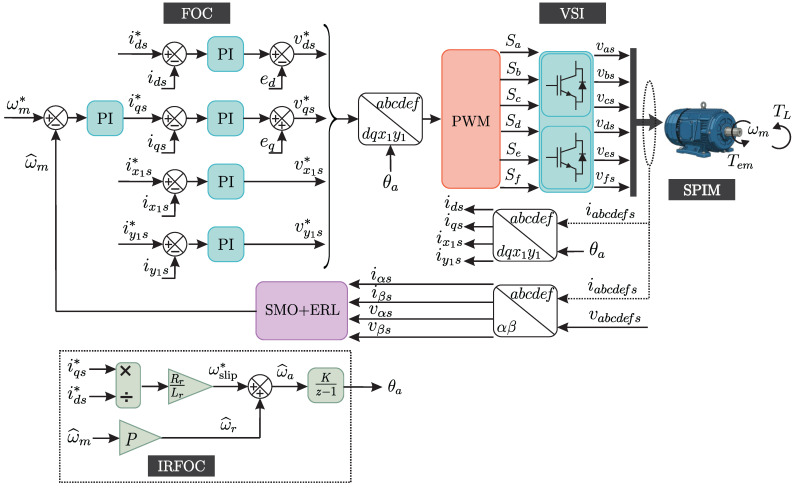
IRFOC scheme for an asymmetrical SPIM with isolated neutrals.

As shown in Figure 2, the IRFOC strategy applied to the asymmetrical SPIM is based on the transformation of stator currents from the stationary (α–β) reference frame into a synchronously rotating (d–q) frame. By aligning the *d*-axis with the rotor flux vector, the direct-axis current component governs the flux level, whereas the quadrature-axis component controls the electromagnetic torque. This decoupling requires the angular position of the rotating reference frame, denoted as $\theta_{a}$, to be continuously updated.

In sensorless operation, $\theta_{a}$ is not obtained from a mechanical position sensor. Instead, it is reconstructed from the estimated electrical rotor speed provided by the SMO + ERL and the slip angular frequency required by the IRFOC scheme. The observer estimates the electrical rotor speed ${\hat{\omega }}_{r}$ in the stationary (α–β) subspace, and the corresponding estimated mechanical speed is computed as: 

(19) $${\hat{\omega }}_{m}=\frac{{\hat{\omega }}_{r}}{P},\;$$

This estimated speed is used as feedback in the outer speed-control loop, whose output defines the reference quadrature-axis current $i_{qs}^{*}$. The direct-axis current reference $i_{ds}^{*}$ is kept constant to impose the rotor-flux orientation.

The slip angular frequency is then calculated from the rotor parameters and current references according to the standard IRFOC formulation [32,37]:

(20) $$\omega_{\mathrm{slip}}^{*}=\frac{R_{r}}{L_{r}}\frac{i_{qs}^{*}}{i_{ds}^{*}},$$

The estimated synchronous angular speed used to generate the rotating reference frame is therefore obtained as:

(21) $${\hat{\omega }}_{a}={\hat{\omega }}_{r}+\omega_{\mathrm{slip}}^{*}.$$

Finally, the angular position required by the Park and inverse Park transformations is computed by integrating ${\hat{\omega }}_{a}$:

(22) $$\theta_{a}\left(t\right)=\int_{0}^{t}{\hat{\omega }}_{a}\left(t^{\prime }\right)\;dt^{\prime }$$

The Park transformation applied to the stator current components is given by:

(23) $$\left[\begin{array}{c} i_{ds}\\ i_{qs} \end{array}\right]=\left[\begin{array}{cc} \cos\theta_{a} & \sin\theta_{a}\\ -\sin\theta_{a} & \cos\theta_{a} \end{array}\right]\left[\begin{array}{c} i_{\alpha s}\\ i_{\beta s} \end{array}\right],$$

where $\theta_{a}$ denotes the angular position of the rotating reference frame [37].

The speed regulation loop is implemented using a proportional–integral (PI) controller, whose output defines the reference for the *q*-axis stator current. This loop compensates for load disturbances and ensures accurate tracking of the speed reference. The control law of the speed controller can be expressed as:

(24) $$i_{qs}^{*}=K_{p}\left(\omega_{m}^{*}-{\hat{\omega }}_{m}\right)+K_{i}\int \left(\omega_{m}^{*}-{\hat{\omega }}_{m}\right)dt,\;$$

where $\omega_{m}^{*}$ is the reference mechanical speed, ${\hat{\omega }}_{m}$ corresponds to the estimated speed, $K_{p}$ and $K_{i}$ are the PI controller gains.

The inner current control loops regulate the *d*- and *q*-axis stator currents using PI controllers with decoupling terms that compensate for cross-coupling effects. The reference *d*-axis current is kept constant at its nominal value to guarantee proper magnetization of the machine. In addition, the current references in the (x–y) subspace are set to zero in order to suppress non-torque-producing harmonic components and minimize copper losses, exploiting the isolated neutral configuration of the SPIM.

### 3.2. Sliding Mode Observer with Exponential Reaching Law (SMO + ERL)

The proposed observer is formulated in the stationary (α–β) subspace, where the electromechanical energy conversion takes place, and preserves the conventional SMO state structure derived from the induction machine model expressed in the stationary reference frame. The observer employs measured stator currents together with estimated rotor flux components in the (α–β) subspace to estimate the rotor speed within the IRFOC scheme. Unlike classical SMO implementations, which typically require a LPF to reconstruct the equivalent control signal, the proposed approach incorporates an ERL-based adaptive gain mechanism to obtain the speed estimation signal directly from the switching function without any auxiliary filtering stage. Consequently, the proposed formulation avoids the phase delay associated with LPF-based approaches while improving the smoothness of the estimated signals. The estimated speed is then used for both feedback and synchronous reference-frame generation within the IRFOC structure, whereas the non-torque-producing (x–y) current components are regulated to zero in order to reduce copper losses.

#### 3.2.1. Observer State Equations

The state equations describing the electrical dynamics of the induction machine are expressed as:

(25) $$\begin{array}{cc} {\dot{\psi }}_{\alpha r} & =-a_{5}\psi_{\alpha r}-\omega_{r}\psi_{\beta r}+a_{4}i_{\alpha s}, \end{array}$$

(26) $$\begin{array}{cc} {\dot{\psi }}_{\beta r} & =-a_{5}\psi_{\beta r}+\omega_{r}\psi_{\alpha r}+a_{4}i_{\beta s}, \end{array}$$

(27) $$\begin{array}{cc} {\dot{i}}_{\alpha s} & =a_{2}\psi_{\alpha r}+a_{3}\omega_{r}\psi_{\beta r}-a_{1}i_{\alpha s}+a_{6}v_{\alpha s}, \end{array}$$

(28) $$\begin{array}{cc} {\dot{i}}_{\beta s} & =a_{2}\psi_{\beta r}-a_{3}\omega_{r}\psi_{\alpha r}-a_{1}i_{\beta s}+a_{6}v_{\beta s}, \end{array}$$

where $a_{1},\ldots ,a_{6}$ are positive coefficients defined from the electrical parameters of the machine as:  

(29) $$\begin{array}{cc} a_{1} & =\frac{R_{s}}{\sigma L_{s}}+\frac{M^{2}}{\sigma L_{s}L_{r}\tau_{r}},\;a_{2}=\frac{M}{\sigma L_{s}L_{r}\tau_{r}},\;a_{3}=\frac{M}{\sigma L_{s}L_{r}},\\ a_{4} & =\frac{M}{\tau_{r}},\;a_{5}=\frac{1}{\tau_{r}},\;a_{6}=\frac{1}{\sigma L_{s}}, \end{array}$$

with $\tau_{r}=L_{r}/R_{r}$ and $\sigma =1-M^{2}/\left(L_{s}L_{r}\right)$. The corresponding observer dynamics for the estimated rotor flux and stator currents are defined as:

(30) $$\begin{array}{cc} {\dot{\hat{\psi }}}_{\alpha r} & =-a_{5}{\hat{\psi }}_{\alpha r}-{\hat{\omega }}_{r}{\hat{\psi }}_{\beta r}+a_{4}i_{\alpha s}, \end{array}$$

(31) $$\begin{array}{cc} {\dot{\hat{\psi }}}_{\beta r} & =-a_{5}{\hat{\psi }}_{\beta r}+{\hat{\omega }}_{r}{\hat{\psi }}_{\alpha r}+a_{4}i_{\beta s}, \end{array}$$

(32) $$\begin{array}{cc} {\dot{\hat{i}}}_{\alpha s} & =a_{2}{\hat{\psi }}_{\alpha r}+a_{3}{\hat{\omega }}_{r}{\hat{\psi }}_{\beta r}-a_{1}{\hat{i}}_{\alpha s}+a_{6}v_{\alpha s}, \end{array}$$

(33) $$\begin{array}{cc} {\dot{\hat{i}}}_{\beta s} & =a_{2}{\hat{\psi }}_{\beta r}-a_{3}{\hat{\omega }}_{r}{\hat{\psi }}_{\alpha r}-a_{1}{\hat{i}}_{\beta s}+a_{6}v_{\beta s}. \end{array}$$

#### 3.2.2. Estimation Error Dynamics


(34) $$\begin{array}{cc} {\tilde{i}}_{\alpha s}={\hat{i}}_{\alpha s}-i_{\alpha s}\;,\; & \;{\tilde{i}}_{\beta s}={\hat{i}}_{\beta s}-i_{\beta s}\;,\\ {\tilde{\psi }}_{\alpha r}={\hat{\psi }}_{\alpha r}-\psi_{\alpha r}\;,\; & \;{\tilde{\psi }}_{\beta r}={\hat{\psi }}_{\beta r}-\psi_{\beta r}\;,\\ {\tilde{\omega }}_{r}={\hat{\omega }}_{r}-\omega_{r}. & \; \end{array}$$

By subtracting the plant equations from the observer equations, the current estimation error dynamics can be obtained as:

(35) $$\begin{array}{cc} {\dot{\tilde{\psi }}}_{\alpha r} & =-a_{5}{\tilde{\psi }}_{\alpha r}-{\tilde{\omega }}_{r}{\hat{\psi }}_{\beta r}-\omega_{r}{\tilde{\psi }}_{\beta r},\; \end{array}$$

(36) $$\begin{array}{cc} {\dot{\tilde{\psi }}}_{\beta r} & =-a_{5}{\tilde{\psi }}_{\beta r}+{\tilde{\omega }}_{r}{\hat{\psi }}_{\alpha r}-\omega_{r}{\tilde{\psi }}_{\alpha r},\; \end{array}$$

(37) $$\begin{array}{cc} {\dot{\tilde{i}}}_{\alpha s} & =-a_{1}{\tilde{i}}_{\alpha s}+a_{2}{\tilde{\psi }}_{\alpha r}+a_{3}\omega_{r}{\tilde{\psi }}_{\beta r}+a_{3}{\tilde{\omega }}_{r}{\hat{\psi }}_{\beta r}, \end{array}$$

(38) $$\begin{array}{cc} {\dot{\tilde{i}}}_{\beta s} & =-a_{1}{\tilde{i}}_{\beta s}+a_{2}{\tilde{\psi }}_{\beta r}-a_{3}\omega_{r}{\tilde{\psi }}_{\alpha r}-a_{3}{\tilde{\omega }}_{r}{\hat{\psi }}_{\alpha r}. \end{array}$$

## 4. Stability Analysis of the SMO + ERL Without LPF

This section establishes sufficient stability conditions for the proposed SMO + ERL speed observer when the discontinuous switching function is implemented directly using the sign operator, thereby avoiding an auxiliary LPF. The derivation follows the same Lyapunov-based framework commonly adopted in SMO analyses for multiphase induction machines, where the current estimation error is first studied and subsequently the rotor flux estimation error is examined under sliding mode [24].

### 4.1. Sliding Surface Definition

To enforce convergence of the current estimation errors, the sliding surface is defined as:

(39) $$S_{bn}={\tilde{i}}_{\beta s}{\hat{\psi }}_{\alpha r}-{\tilde{i}}_{\alpha s}{\hat{\psi }}_{\beta r}.\;$$

This surface represents a weighted cross-product between the stator current error and the estimated rotor flux vector, thereby promoting both current-error reduction and proper alignment of the rotor flux and stator current in the (α–β) plane, consistent with flux-oriented control. The condition $S_{bn}\to 0$ ensures current error minimization and correct flux-current alignment under rotor-flux oriented control.

### 4.2. ERL Speed Adaptation Without LPF

The proposed observer directly uses the discontinuous sign function combined with the exponential reaching gain:

(40) $${\hat{\omega }}_{r}=K_{S_{bn}}\;\mathrm{sign}\left(S_{bn}\right),\;$$

with the adaptive gain defined as:

(41) $$K_{S_{bn}}=K_{\min}+\left(K_{\max}-K_{\min}\right)\left(1-e^{-\lambda |S_{bn}|}\right),\;K_{\max}\geq K_{\min}>0,\;\lambda >0,\;$$

where $K_{\min}$ and $K_{\max}$ are the minimum and maximum observer gains, and λ a curvature parameter governing how fast $K_{S_{bn}}$ transitions from $K_{\min}$ to $K_{\max}$ as $|S_{bn}|$ increases.

The corresponding speed estimation error becomes:

(42) $${\tilde{\omega }}_{r}=K_{S_{bn}}\mathrm{sign}\left(S_{bn}\right)-\omega_{r}.\;$$

This formulation preserves the robustness properties of sliding-mode observers while removing the delay and bandwidth limitations typically introduced by LPF. In addition, the gain scheduling mechanism provides aggressive correction for large estimation errors and reduced switching effort as the trajectory approaches the sliding manifold.

**Figure 3:**
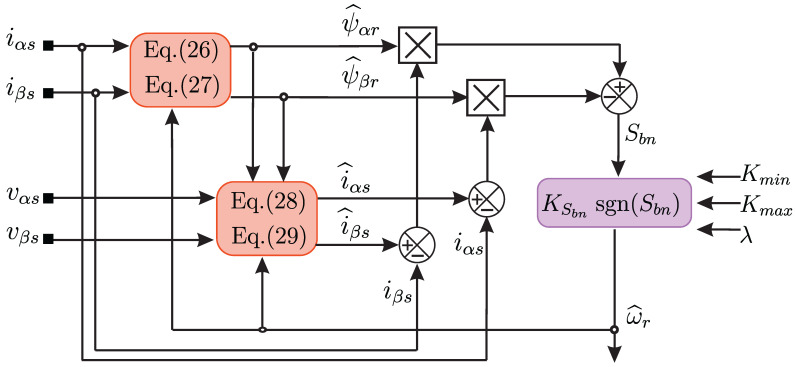
Block diagram of the SMO + ERL acting directly on the estimated electrical speed.

The estimated rotor speed ${\hat{\omega }}_{r}$ obtained from the SMO + ERL is fed back into both the speed control loop and the reference frame transformation, enabling fully sensorless IRFOC operation. The modular nature of the observer allows it to be integrated with different current control strategies without modifying the overall control structure. A schematic representation of the proposed SMO + ERL implementation is presented in Figure 3.

After the mathematical definition of the sliding variable $S_{bn}$ and the adaptive ERL gain $K_{S_{bn}}$, Algorithm 1 summarizes the real-time implementation sequence of the proposed SMO + ERL-based sensorless control strategy. The algorithm is executed at each sampling period and includes the observer-state update, adaptive reaching-gain computation, rotor-speed estimation, synchronous reference-frame generation, current-control action, inverse transformations, and CB-PWM generation.
**Algorithm 1** Proposed SMO + ERL-based sensorless control algorithm.  1:**Initialization:** Set $T_{s}$, $K_{\min}$, $K_{\max}$, λ, $i_{ds}^{*}$, and initialize ${\hat{i}}_{\alpha s}$, ${\hat{i}}_{\beta s}$, ${\hat{\psi }}_{\alpha r}$, ${\hat{\psi }}_{\beta r}$, ${\hat{\omega }}_{r}$, and $\theta_{a}$.  2:**while** the drive is enabled **do**  3:      **Acquire signals:** Measure the stator phase currents and reconstruct the unmeasured phase current in each three-phase set.  4:      **VSD transformation:** Transform the measured currents and voltage references into the (α–β) and (x–y) subspaces.  5:      **Observer update:** Update ${\hat{i}}_{\alpha s}$, ${\hat{i}}_{\beta s}$, ${\hat{\psi }}_{\alpha r}$, and ${\hat{\psi }}_{\beta r}$ using the discretized SMO equations.  6:      **Sliding variable:** Compute the current estimation errors and evaluate $S_{bn}$.  7:      **ERL adaptive gain:** Compute$$K_{S_{bn}}=K_{\min}+\left(K_{\max}-K_{\min}\right)\left(1-e^{-\lambda |S_{bn}|}\right).$$  8:      **Speed estimation:** Estimate the electrical rotor speed as ${\hat{\omega }}_{r}=K_{S_{bn}}\mathrm{sign}\left(S_{bn}\right)$ and compute ${\hat{\omega }}_{m}={\hat{\omega }}_{r}/P$.  9:      **Speed control:** Use ${\hat{\omega }}_{m}$ in the speed PI controller to generate $i_{qs}^{*}$; set $i_{ds}^{*}=\mathrm{const}$, $i_{xs}^{*}=0$, and $i_{ys}^{*}=0$.10:      **Synchronous angle generation:** Compute $\omega_{\mathrm{slip}}^{*}$, obtain ${\hat{\omega }}_{a}={\hat{\omega }}_{r}+\omega_{\mathrm{slip}}^{*}$, and update $\theta_{a}$.11:      **Park transformation:** Transform the measured (α–β) currents into the synchronous (d–q) frame using $\theta_{a}$ to obtain $i_{ds}$ and $i_{qs}$.12:      **Current control:** Execute the (d–q) and (x–y) current controllers to obtain $v_{ds}^{*}$, $v_{qs}^{*}$, $v_{xs}^{*}$, and $v_{ys}^{*}$.13:      **Inverse transformations:** Apply the inverse Park and inverse VSD transformations to obtain the six-phase voltage references.14:      **PWM generation:** Generate the switching signals for the six-phase VSI using CB-PWM.15:      Wait until the next sampling instant.16:**end while**

### 4.3. Stability of the Current Estimation Error (Lyapunov Analysis)

Define the same positive Lyapunov candidate used in classical SMO analyses:

(43) $$I=\frac{1}{2}\left({\tilde{i}}_{\alpha s}^{2}+{\tilde{i}}_{\beta s}^{2}\right),\;I\geq 0.$$

Using the current estimation error dynamics (37) and (38):

(44) $$\begin{array}{cc} \dot{I} & ={\tilde{i}}_{\alpha s}{\dot{\tilde{i}}}_{\alpha s}+{\tilde{i}}_{\beta s}{\dot{\tilde{i}}}_{\beta s}=-2a_{1}I-a_{3}{\tilde{\omega }}_{r}S_{bn}-a_{3}\omega_{r}\left(S_{bn}-S_{n}\right)+a_{2}S_{c},\; \end{array}$$

where the following auxiliary cross terms are defined:

(45) $$\begin{array}{cc} S_{bn}-S_{n} & ={\tilde{i}}_{\beta s}{\tilde{\psi }}_{\alpha r}-{\tilde{i}}_{\alpha s}{\tilde{\psi }}_{\beta r}\;, \end{array}$$

(46) $$\begin{array}{cc} S_{n} & ={\tilde{i}}_{\beta s}\psi_{\alpha r}-{\tilde{i}}_{\alpha s}\psi_{\beta r}, \end{array}$$

(47) $$\begin{array}{cc} S_{c} & ={\tilde{i}}_{\alpha s}{\tilde{\psi }}_{\alpha r}+{\tilde{i}}_{\beta s}{\tilde{\psi }}_{\beta r}. \end{array}$$

Substituting (42) into (44), and using the property $\mathrm{sgn}\left(S_{bn}\right)S_{bn}=\left|S_{bn}\right|$, yields

(48) $$\dot{I}=-2a_{1}I+a_{3}|S_{bn}|\left(|\omega_{r}|-K_{S_{bn}}\right)+a_{3}|\omega_{r}\left|\right|S_{bn}-S_{n}|+a_{2}\left|S_{c}\right|.$$

A sufficient condition ensuring $\dot{I}<0$ is obtained by enforcing a strict dominance of the ERL gain over the remaining terms:

(49) $$K_{S_{bn}}|S_{bn}\left|>\right|\omega_{r}\left|\right|S_{n}|-\frac{2a_{1}}{a_{3}}I+\frac{a_{2}}{a_{3}}\left|S_{c}\right|+\varepsilon ,\;\varepsilon >0,$$

which generalizes the classical constant-gain inequality by replacing $K_{s}$ in [24] with the state-dependent $K_{S_{bn}}$.

Because $K_{S_{bn}}$ is bounded as $K_{\min}\leq K_{S_{bn}}\leq K_{\max}$, a conservative global design can be stated as:

(50) $$K_{\max}\geq \omega_{\max}+\Delta_{K},\;\Delta_{K}>0,$$

where $\omega_{\max}$ is the maximum expected electrical speed and $\Delta_{K}$ is a robustness margin covering modeling uncertainties. In contrast to the LPF-based SMO, the ERL law yields a smooth estimate directly, avoiding additional phase lag introduced by filtering.

### 4.4. Stability of the Rotor Flux Estimation Error Under Sliding

Once the system reaches the sliding regime, $S_{bn}=0$ and ${\dot{S}}_{bn}=0$. Under this condition, the equivalent control associated with the discontinuous switching term coincides with the actual rotor speed information [24].

Taking the derivative of $S_{bn}$ in (39) and substituting (36) and (37), one obtains (after algebraic manipulation) an expression of the form:

(51) $${\dot{S}}_{bn}=a_{2}\left({\tilde{\psi }}_{\beta r}{\hat{\psi }}_{\alpha r}-{\tilde{\psi }}_{\alpha r}{\hat{\psi }}_{\beta r}\right)-a_{3}{\hat{\omega }}_{r}\left({\hat{\psi }}_{\alpha r}^{2}+{\hat{\psi }}_{\beta r}^{2}\right)+a_{3}\omega_{r}\left(\psi_{\alpha r}{\hat{\psi }}_{r\alpha }+\psi_{\beta r}{\hat{\psi }}_{\beta r}\right),\;$$

Under sliding, imposing ${\dot{S}}_{bn}\approx 0$ yields an equivalent speed estimate:

(52) $${\hat{\omega }}_{r,\mathrm{eq}}\approx \omega_{r}\;\frac{\psi_{\alpha r}{\hat{\psi }}_{\alpha r}+\psi_{\beta r}{\hat{\psi }}_{\beta r}}{{\hat{\psi }}_{\alpha r}^{2}+{\hat{\psi }}_{\beta r}^{2}}+a_{5}\;\frac{{\tilde{\psi }}_{\beta r}{\hat{\psi }}_{\alpha r}-{\tilde{\psi }}_{\alpha r}{\hat{\psi }}_{\beta r}}{{\hat{\psi }}_{\alpha r}^{2}+{\hat{\psi }}_{\beta r}^{2}},$$

which matches the classical structure and remains valid provided that ${\hat{\psi }}_{r\alpha }^{2}+{\hat{\psi }}_{r\beta }^{2}$ is bounded away from zero (nonzero magnetization).

Substituting (52) into (35) and (36) yields a linear time-varying system in:

(53) $${\dot{\tilde{\psi }}}_{r}=A_{\psi }\left(t\right)\;{\tilde{\psi }}_{r},$$

(54) $$A_{\psi }=\left[\begin{array}{cc} \frac{-a_{5}{\hat{\psi }}_{\alpha r}^{\;2}+\omega_{r}{\hat{\psi }}_{\alpha r}{\hat{\psi }}_{\beta r}}{{\hat{\psi }}_{\alpha r}^{\;2}+{\hat{\psi }}_{\beta r}^{\;2}} & \frac{-\omega_{r}{\hat{\psi }}_{\alpha r}^{\;2}-a_{5}{\hat{\psi }}_{\alpha r}{\hat{\psi }}_{\beta r}}{{\hat{\psi }}_{\alpha r}^{\;2}+{\hat{\psi }}_{\beta r}^{\;2}}\\ \frac{\omega_{r}{\hat{\psi }}_{\beta r}^{\;2}-a_{5}{\hat{\psi }}_{\alpha r}{\hat{\psi }}_{\beta r}}{{\hat{\psi }}_{\alpha r}^{\;2}+{\hat{\psi }}_{\beta r}^{\;2}} & \frac{-a_{5}{\hat{\psi }}_{\beta r}^{\;2}-\omega_{r}{\hat{\psi }}_{\alpha r}{\hat{\psi }}_{\beta r}}{{\hat{\psi }}_{\alpha r}^{\;2}+{\hat{\psi }}_{\beta r}^{\;2}} \end{array}\right]$$

where ${\tilde{\psi }}_{r}={\left[{\tilde{\psi }}_{r\alpha }\;{\tilde{\psi }}_{r\beta }\right]}^{T}$ and $A_{\psi }$ is a time-varying matrix dependent on rotor speed and flux magnitude. The characteristic structure is the same as in classical SMO analyses, where one eigenvalue is strictly negative (related to $-a_{5}$), ensuring attenuation of the flux estimation error, while the remaining dynamics remain bounded under normal operating conditions.

#### Flux-Error Lyapunov Function

To keep the same Lyapunov philosophy (separate current and flux analyses), define:

(55) $$V_{\psi }=\frac{1}{2}\left({\tilde{\psi }}_{\alpha r}^{2}+{\tilde{\psi }}_{\beta r}^{2}\right),\;V_{\psi }\geq 0.$$

Using (35) and (36):

(56) $$\begin{array}{cc} {\dot{V}}_{\psi } & ={\tilde{\psi }}_{\alpha r}{\dot{\tilde{\psi }}}_{\alpha r}+{\tilde{\psi }}_{\beta r}{\dot{\tilde{\psi }}}_{\beta r}=-a_{5}\left({\tilde{\psi }}_{\alpha r}^{2}+{\tilde{\psi }}_{\beta r}^{2}\right)+{\tilde{\omega }}_{r}\left({\tilde{\psi }}_{\beta r}{\hat{\psi }}_{\alpha r}-{\tilde{\psi }}_{\alpha r}{\hat{\psi }}_{\beta r}\right). \end{array}$$

Under sliding, the residual ${\tilde{\omega }}_{r}$ becomes small (ideal case: ${\tilde{\omega }}_{r}\to 0$), and the dominant term in (56) is $-a_{5}{∥{\tilde{\psi }}_{r}∥}^{2}$, which implies exponential decay of the flux error. Therefore:

(57) $${\dot{V}}_{\psi }\leq -2a_{5}V_{\psi }+|{\tilde{\omega }}_{r}|\;∥{\tilde{\psi }}_{r}∥\;∥{\hat{\psi }}_{r}∥.$$

Hence, in the ideal sliding regime,

(58) $${\tilde{\psi }}_{\alpha r}\to 0,\;{\tilde{\psi }}_{\beta r}\to 0,\;{\tilde{\omega }}_{r}\to 0.$$

This proves asymptotic convergence of the rotor-flux estimation error and the accuracy of speed reconstruction in the sense of equivalent control.

Finally, from the preceding Lyapunov-based analysis of the current and rotor flux estimation errors, it can be concluded that the proposed SMO + ERL observer without LPF guarantees global reaching of the sliding surface and asymptotic stability of the estimation errors under suitable gain selection. The ERL-based adaptation law ensures that the sliding variable $S_{bn}$ is driven to zero in finite time, while the rotor flux estimation error dynamics remain exponentially stable once the ideal sliding regime is established.

Unlike conventional SMO implementations that employ discontinuous switching followed by LPF, the proposed ERL formulation directly applies the switching action through a gain-scheduled discontinuous law. As a result, additional phase delay is eliminated, and the natural damping of the estimation error dynamics is preserved. The parameters $K_{\min}$ and $K_{\max}$ explicitly determine the local and global convergence properties, whereas the curvature coefficient λ governs how rapidly the adaptive gain transitions from low-error operation to aggressive correction under large estimation mismatches. Consequently, the reaching speed and the robustness margin can be tuned systematically.

Furthermore, stability requires that the rotor flux magnitude remains persistently nonzero, ensuring that the denominator in the equivalent speed expression does not vanish. This condition is naturally satisfied in rotor-flux-oriented control under normal operating conditions. In discrete-time implementation, backward Euler discretization preserves the dissipative structure of the continuous-time Lyapunov function, ensuring that numerical integration does not destabilize the observer provided that the sampling period $T_{s}$ is sufficiently small relative to the rotor electrical time constant.

Finally, when integrated within the outer FOC loop, the absence of a LPF improves phase margin and dynamic tracking capability. Since the ERL-based speed estimate is obtained without introducing filtering delay, the overall closed-loop system is expected to exhibit a faster transient response while maintaining bounded estimation errors.

Therefore, under appropriate gain selection ($K_{\max}$ sufficiently large for global reaching, $K_{\min}>0$ for local correction, and λ>0 for adequate gain scheduling), the proposed SMO + ERL observer guarantees:

(59) $${\tilde{i}}_{\alpha ,\beta }\left(t\right)\to 0,\;{\tilde{\psi }}_{r\alpha ,\beta }\left(t\right)\to 0,\;{\tilde{\omega }}_{r}\left(t\right)\to 0,$$

in the ideal case, and practical convergence to a small neighborhood of the origin in the presence of bounded disturbances and implementation nonidealities.

## 5. Results

The results are organized into two subsections: simulation results and experimental results. First, a set of tests is performed in a simulation environment to evaluate the performance of the proposed sensorless control. Then, experimental results obtained from a laboratory setup are presented. In both cases, the classical LPF-based SMO and the proposed SMO + ERL were evaluated using the same SPIM parameters, IRFOC structure, current controllers, sampling frequency, discretization method, magnetizing current reference and operating conditions. Therefore, the comparison focuses on the speed-estimation mechanism itself: a fixed-gain SMO with LPF versus an adaptive-gain SMO + ERL without LPF. The observer parameters used in both the simulation and experimental tests are summarized in Table 1.

### 5.1. Simulation Results

This section provides a simulation-based validation using the MATLAB/Simulink R2023b platform. The developed model captures the essential electrical and mechanical dynamics of the proposed SPIM drive, and all simulations are performed using the common implementation settings and observer parameters reported in Table 1, ensuring adequate time resolution for both dynamic transients and steady-state operating conditions. The set of electrical and mechanical parameters of the SPIM used in the simulations is reported in Table 2, including the rated operating values, stator and rotor resistances, mutual and leakage inductances, as well as the inertia and friction coefficients. These values are selected to represent the same characteristics of the machine used in the experimental test bench setup, ensuring consistency between simulated and measured results.

**Figure 4:**
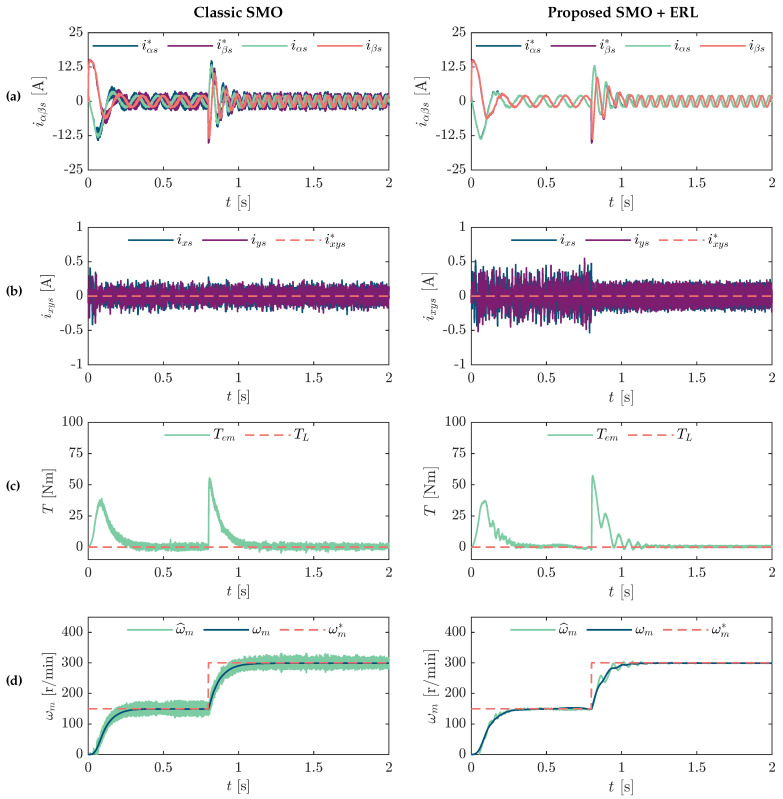
Comparative performance of the classical SMO and the proposed SMO + ERL under no-load conditions when the speed reference is increased from 150 r/min to 300 r/min at t=0.8 s. (**a**) Stator current $i_{\alpha \beta s}$, (**b**) stator current $i_{xys}$, (**c**) electromagnetic torque response, (**d**) mechanical speed.

Figure 4 presents the comparative performance of the classical SMO and the proposed SMO + ERL under no-load conditions when the speed reference is increased from 150 r/min to 300 r/min at t=0.8 s. Figure 4a,b show the evolution of the stator currents in the (α–β) and (x–y) subspaces from the transient interval to steady-state operation. In the (α–β) subspace, the current responses remain well behaved for both techniques, thereby preserving the torque-producing dynamics after the speed step. Nevertheless, a noticeable reduction in current ripple is achieved when the proposed SMO + ERL is applied.

A comparison of the (x–y) currents further indicates that the proposed SMO + ERL produces a slight increase in these components at low speed. This behavior can be attributed to the adaptive gain action introduced by the ERL, which reinforces the observer correction in operating regions where the back-EMF is low and the speed estimation task becomes more demanding. As a consequence, a minor increase in the non-torque-producing current components is observed; however, this effect is accompanied by a significant reduction in the torque and speed estimation errors, as shown in Figure 4c,d. In both cases, the observers track the speed variation and maintain stable operation during the transient. However, the proposed SMO + ERL exhibits clearly superior steady-state accuracy with respect to the classical SMO. In particular, the steady-state speed estimation error is approximately 22.3 r/min for the classical SMO, whereas it is reduced to approximately 5.29 r/min with the SMO + ERL, confirming the benefit of the ERL for improving low-speed estimation performance. Therefore, Figure 4 shows that the SMO + ERL achieves a more favorable compromise between speed estimation accuracy and current quality than the conventional fixed-gain SMO, especially in the low-speed region.

**Figure 5:**
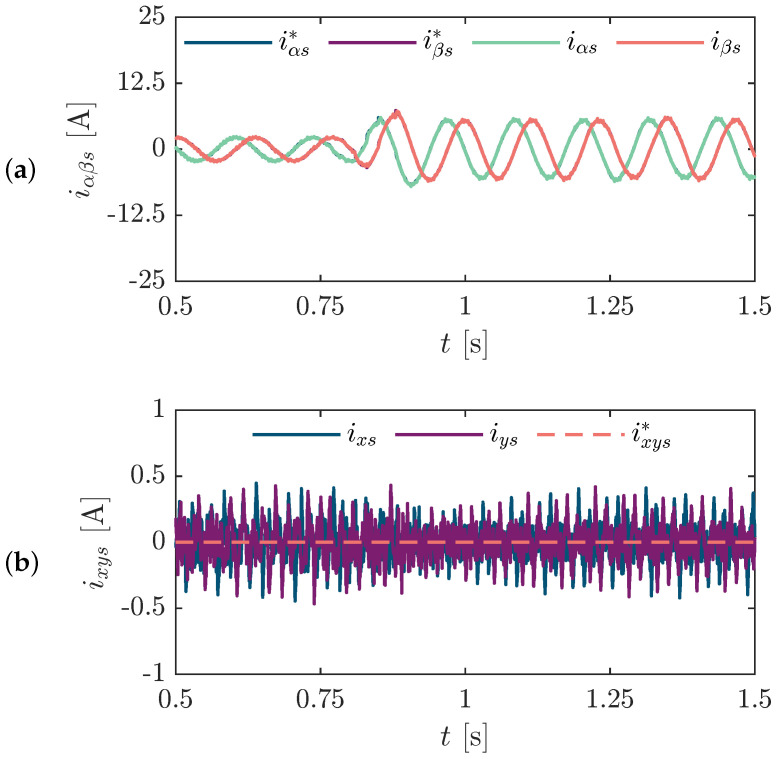

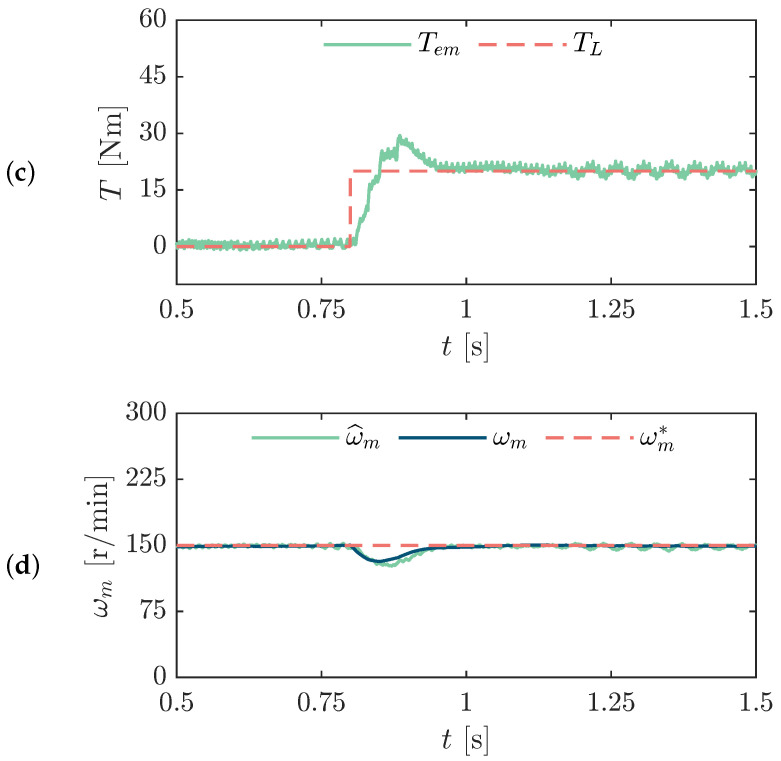
Simulation results obtained for the proposed SMO + ERL at 150 r/min with a 20 Nm load torque step applied at t=0.8 s. (**a**) Stator current $i_{\alpha \beta s}$, (**b**) stator current $i_{xys}$, (**c**) electromagnetic torque response, (**d**) mechanical speed.

Furthermore, to assess the load-disturbance response of the proposed observer, Figure 5 shows the simulated behavior of the SMO + ERL under a load torque step of 20Nm applied at t=0.8s, while the speed reference is kept at 150r/min. After the load step, the electromagnetic torque rapidly increases, showing a short overshoot due to the corrective action of the speed-control loop, whereas the actual and estimated mechanical speeds exhibit a transient deviation before returning to the reference value. The stator currents in the (α–β) subspace increase according to the torque demand, while the (x–y) currents remain bounded around zero. These results confirm that the proposed SMO + ERL maintains stable sensorless operation under load-disturbance conditions.

The simulation results discussed above provide initial validation of the proposed SMO + ERL under controlled operating conditions, highlighting its fast convergence, reduced chattering, and accurate speed-tracking capability. To further assess its practical applicability, the following subsection presents the experimental evaluation conducted on the laboratory test bench, in which the observer’s performance is examined under practical operating conditions.

### 5.2. Experimental Results

**Figure 6:**
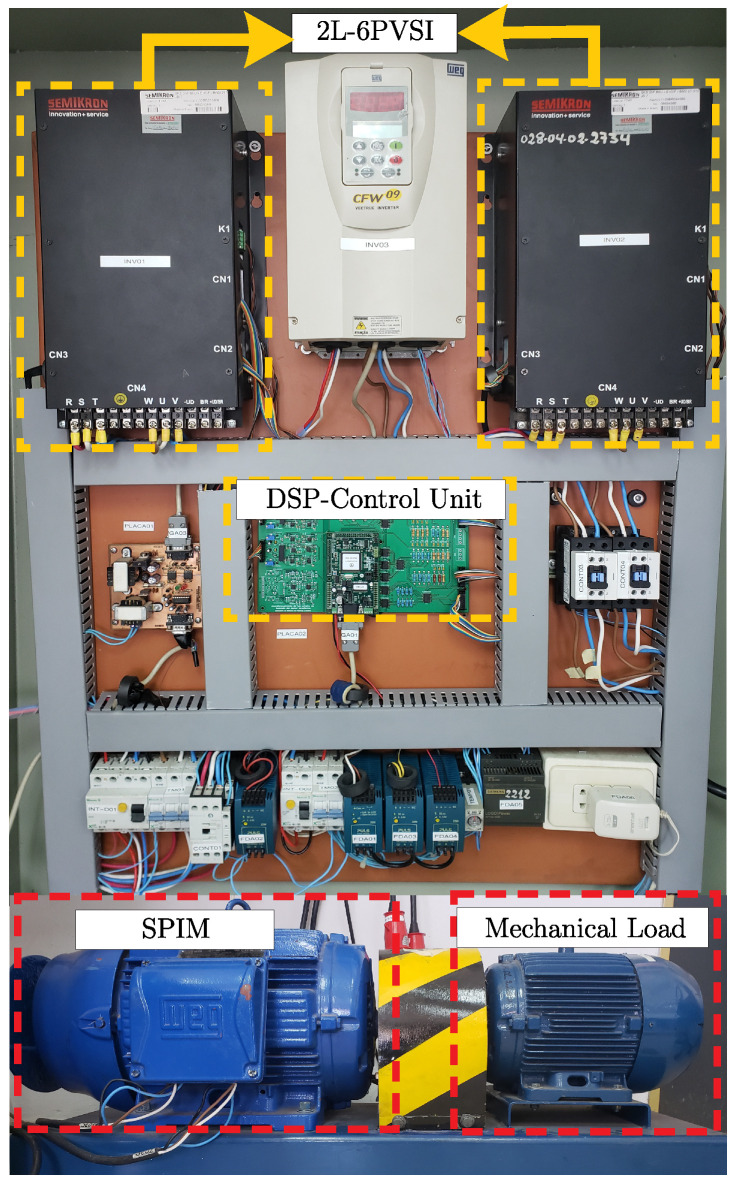
Schematic of the experimental test bench.

The experimental results were obtained from the laboratory test bench shown in Figure 6. The setup consists of an asymmetrical SPIM with isolated neutral points, and its electrical and mechanical parameters are listed in Table 2.

The SPIM is supplied by a six-phase VSI assembled from two three-phase SEMIKRON SKS 35F modules. In each three-phase set, two stator currents are acquired by Hall-effect transducers (LEM LA-55), whereas the third current is reconstructed analytically from the measured signals. The control algorithm and the observers were implemented in discrete time using Euler discretization, following the common settings summarized in Table 1. Experimental validation was performed on an MSK28335 development board incorporating a TMS320LF28335 floating-point DSP.

Under this experimental arrangement, the rotor mechanical speed is measured using an optical incremental encoder that produces a pulse sequence proportional to the shaft position. In the present setup, a DFS60B-S1PB10000 encoder with a resolution of 10,000 ppr is employed. The corresponding pulse train is processed through the dedicated quadrature-encoder interface on the TMS320F28335 DSP, thereby enabling reliable speed reconstruction for validation. It should be emphasized that this encoder-based measurement is used exclusively as a reference signal to assess the accuracy of the proposed estimator during the experimental evaluation; therefore, it is not fed back into the control loop, which preserves the sensorless nature of the implemented control strategy.

Subsequently, the experimental data obtained from the tests are processed and the corresponding figures are generated in the MATLAB environment to ensure consistent analysis and clear visualization of the obtained results.

#### 5.2.1. Figures of Merit

The accuracy of the proposed SMO + ERL is quantified through the Root Mean Square Error (RMSE), computed between the current references and the experimentally measured stator currents in each observable subspace. This metric provides a compact indicator of the tracking quality by emphasizing both the amplitude and persistence of the current error. The RMSE is defined as follows:

(60) $$\mathrm{RMSE}\left(i_{\vartheta s}\right)=\sqrt{\frac{1}{\rho }\sum_{k=k_{0}}^{k=k_{0}+\rho }{\left(i_{\vartheta s}\left(k\right)-i_{\vartheta s}^{*}\left(k\right)\right)}^{2}}\;,$$

In Equation (60), $i_{\vartheta s}^{*}$ denotes the stator current reference and $i_{\vartheta s}$ represents the corresponding measured current for ϑ∈ $\left\{\alpha ,\;\beta ,\;x,\;y\right\}$. The parameter ρ is the number of samples included in the evaluation window, whereas $k_{0}$ defines the initial sample of the steady-state interval over which the error is evaluated. Accordingly, the RMSE enables a consistent comparison of current-regulation performance across all considered subspaces. In addition to this current-based metric, the Mean Value Error (MVE) is introduced to assess the steady-state accuracy of the speed estimation.

The MVE is used as a normalized percentage index and measures the average relative deviation between the reference mechanical speed and its estimated value over the selected observation interval. It is expressed as:

(61) $$\mathrm{MVE}\left({\hat{\omega }}_{m}\right)=\frac{1}{\rho }\sum_{k=k_{0}}^{k=k_{0}+\rho }\left|\frac{\omega_{m}^{*}\left(k\right)-{\hat{\omega }}_{m}\left(k\right)}{\omega_{m}^{*}\left(k\right)}\right|\times 100\%.$$

#### 5.2.2. Steady State and Transient Results

**Figure 7:**
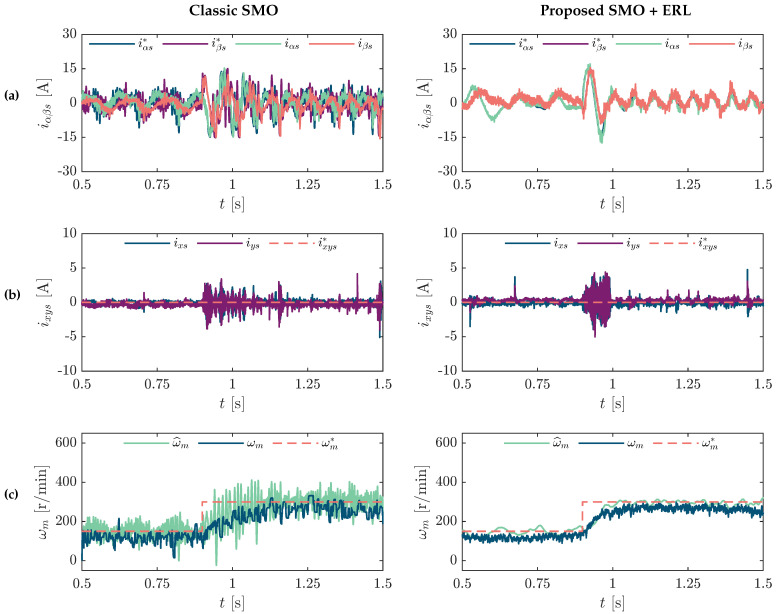
Experimental results obtained for the SMO and the proposed SMO + ERL, under no-load conditions when the speed reference is increased from 150 r/min to 300 r/min at t=0.9 s. (**a**) Stator current $i_{\alpha \beta s}$, (**b**) stator current $i_{xys}$, (**c**) mechanical speed.

The experimental comparison between the classical SMO and the proposed SMO + ERL under no-load conditions, when a speed step is applied from 150 r/min to 300 r/min at approximately t=0.9 s, is presented in Figure 7. The corresponding transient responses of the stator currents and mechanical speed are depicted for both observer configurations.

Figure 7a shows the stator currents in the (α–β) subspace. After the speed transition, the currents obtained with the classical SMO present higher oscillations and waveform distortion during the transient interval. In comparison, the SMO + ERL currents exhibit smoother waveforms and a more regular transition toward steady-state operation. A similar tendency can be observed in Figure 7b, which shows the (x–y) subspace currents. In this case, the classical SMO produces currents with larger fluctuation amplitudes and wider dispersion around zero during the transient, whereas the SMO + ERL currents remain more concentrated around zero with reduced oscillatory behavior throughout the operating interval. The corresponding mechanical speed responses are shown in Figure 7c. Both approaches can track the speed reference after the step variation. However, the classical SMO reaches the reference at approximately t=1.1744 s, while the SMO + ERL achieves convergence earlier, at approximately t=1.0148 s, exhibiting a smoother transient evolution and lower oscillatory behavior.

**Figure 8:**
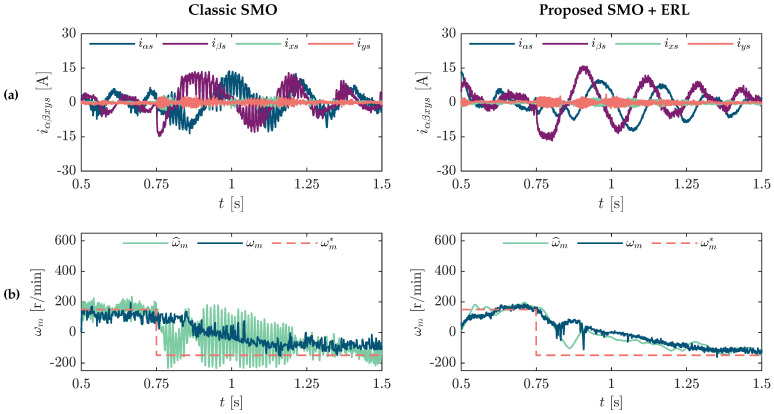
Experimental results for the SMO and the proposed SMO + ERL under no-load conditions during a speed reversal from 150 r/min to −150 r/min. (**a**) Stator current $i_{\alpha \beta xys}$, (**b**) mechanical speed.

In the case of a speed reversal from 150 r/min to −150 r/min, Figure 8 presents the experimental results. The stator current responses corresponding to both observer configurations are depicted in Figure 8a. During the reversal interval, particularly around the zero-speed crossing, the classical SMO currents exhibit pronounced oscillations and waveform distortion. Conversely, the SMO + ERL currents display smoother transitions and reduced oscillatory behavior throughout the transient process. The associated mechanical speed responses are illustrated in Figure 8b. The classical SMO produces a noisier speed trajectory with noticeable oscillations during the inversion process and after the speed crossing. Meanwhile, the SMO + ERL response exhibits a smoother transition and reduced oscillations over the entire response interval, maintaining a more regular speed evolution.

**Figure 9:**
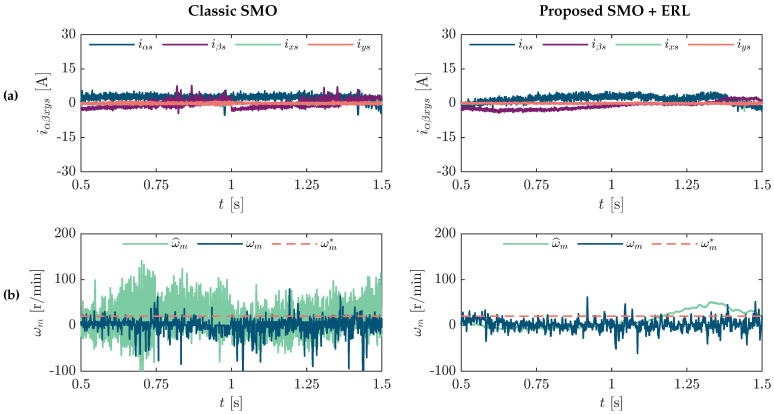
Experimental results for the SMO and the proposed SMO + ERL under no-load conditions during low-speed operation at 20 r/min. (**a**) Stator current $i_{\alpha \beta xys}$, (**b**) mechanical speed.

Additionally, to evaluate the low-speed operating capability of the proposed sensorless strategy, Figure 9 shows the steady-state performance obtained at 20 r/min. The stator currents corresponding to both observer configurations are presented in Figure 9a. Under this operating condition, the classical SMO currents present higher noise and fluctuation levels, while the SMO + ERL currents remain more compact and stable over time. The corresponding mechanical speed responses are depicted in Figure 9b. In this case, the classical SMO estimation contains considerable ripple around the reference value, whereas the SMO + ERL estimation exhibits a smoother and more stable behavior with lower variability in the estimated speed signal.

**Figure 10:**
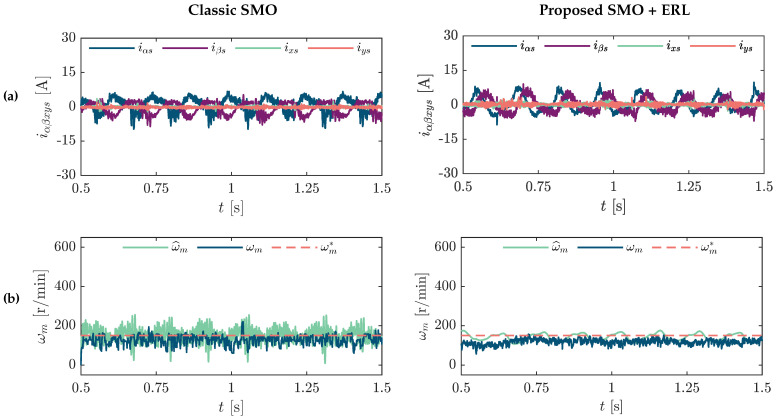
Experimental results under sensorless operation at 150 r/min for the SMO and the proposed SMO + ERL with a load torque of approximately 40 Nm. (**a**) Stator current $i_{\alpha \beta xys}$, (**b**) mechanical speed.

Finally, Figure 10 presents the experimental steady-state performance of the proposed SMO + ERL under loaded operating conditions at 150 r/min with an external load torque of approximately 40 Nm applied to the motor shaft. Figure 10a shows the stator currents in the (α–β) and (x–y) subspaces, where stable current waveforms and reduced oscillatory behavior can be observed during steady-state operation. Additionally, Figure 10b depicts the estimated mechanical speed together with the encoder-measured speed and the reference speed. The results indicate that the proposed observer maintains accurate speed tracking under load conditions, with the estimated speed closely following both the measured and reference signals while preserving a smooth steady-state response.

Although stable sensorless operation is achieved in the experimental tests, the measured current waveforms may still exhibit distortion due to practical implementation aspects. These effects are mainly associated with switching ripple and inverter nonidealities of the two-level six-phase VSI, as well as with the low stator leakage inductance, current-sensor offsets, A/D quantization, residual sliding-mode chattering, and the analytical reconstruction of one phase current in each three-phase set.

## 6. Discussion

### 6.1. Qualitative Performance Under Transient, Low-Speed, and Loaded Operation

The experimental results demonstrate that the proposed SMO + ERL provides stable sensorless operation over a wide operating range and improves the estimation behavior with respect to the conventional LPF-based SMO, particularly under transient and low-speed conditions. During the speed-step tests, the proposed observer exhibits smoother current waveforms in both the (α–β) and (x–y) subspaces, with reduced oscillation amplitude and lower current dispersion around zero. This behavior can be attributed to the combined effect of the ERL-based adaptive gain and the elimination of the LPF stage used in the classical SMO to reconstruct the equivalent control signal. The absence of the LPF avoids the filter-induced phase delay and bandwidth reduction typically introduced by filtering, thereby improving the estimated-speed dynamics during abrupt operating changes and indirectly contributing to smoother current regulation.

The mechanical speed responses further confirm the improved convergence capability of the proposed approach. During large estimation errors, the ERL-based adaptive gain provides a stronger corrective action, which accelerates the reaching phase and improves the transient response. As the trajectory approaches the sliding surface, the adaptive gain decreases its effective correction level, thereby reducing the switching intensity and attenuating the oscillatory behavior in steady state. This mechanism explains the shorter settling time and smoother speed trajectory obtained with the SMO + ERL during the speed-step experiments.

The advantages of the proposed observer are also evident during the speed-reversal tests, particularly around the zero-speed crossing. Under these operating conditions, the back-EMF magnitude is significantly reduced, making the estimation process more sensitive to noise, parameter mismatch, and modeling inaccuracies. In the classical SMO, the limited bandwidth introduced by the LPF contributes to larger oscillations and waveform distortion during the inversion transient. In contrast, the SMO + ERL maintains a smoother current and speed response due to the direct adaptive correction introduced by the exponential reaching law.

The low-speed experimental results at 20r/min further confirm the improved estimation capability of the proposed observer under demanding operating conditions. At very low speed, the reduced back-EMF weakens the information available for sensorless estimation and increases the sensitivity to measurement noise. Although some residual chattering may still appear in practical digital implementations due to the use of the sign(·) function, the ERL-based gain adaptation reduces the effective switching action near the sliding surface, leading to smoother estimated speed signals and more stable current waveforms than those obtained with the classical SMO.

Under loaded operation at 150r/min, the proposed SMO + ERL also preserves stable sensorless operation with a load torque of approximately 40N·m. The loaded experimental results confirm that the proposed observer remains suitable for real-time operation under practical mechanical loading. Nevertheless, the loaded case also shows that the adaptive correction introduced by the ERL may interact differently with the current-regulation dynamics depending on the operating condition, which is further analyzed through the quantitative indicators.

### 6.2. Influence and Selection of the ERL Parameters

The selection of the ERL parameters $K_{\min}$, $K_{\max}$, and λ is directly related to the compromise between convergence speed, chattering attenuation, and estimation-noise sensitivity. These parameters define the adaptive gain $K_{S_{bn}}$ of the proposed reaching law and determine how the observer reacts when the trajectory is far from, or close to, the sliding surface.

The maximum gain $K_{\max}$ defines the correction capability during large transients. It must be sufficiently high to satisfy the reaching requirement associated with the maximum expected electrical rotor speed and to ensure convergence when large estimation errors occur. Increasing $K_{\max}$ accelerates the reaching phase and improves transient convergence; however, excessively high values may amplify measurement noise, unmodeled dynamics, and digital switching effects. Therefore, $K_{\max}$ was selected with a conservative margin relative to the rated electrical rotor speed.

The minimum gain $K_{\min}$ establishes the local correction level near the sliding surface. A very low value of $K_{\min}$ weakens the corrective action around the sliding manifold and may slow down local convergence. Conversely, an excessively high value increases the effective switching activity in steady state, which may lead to larger speed ripple and higher sensitivity to measurement noise. Thus, $K_{\min}$ was selected to maintain sufficient local correction while avoiding excessive steady-state oscillations.

The curvature parameter λ determines the rate at which the adaptive gain transitions from $K_{\min}$ to $K_{\max}$ as the magnitude of the sliding variable $|S_{bn}|$ increases. Larger values of λ produce a faster transition toward $K_{\max}$, resulting in a more aggressive correction during transients. However, this may increase the switching activity and the noise sensitivity of the estimated speed. Smaller values of λ provide smoother estimation signals but slow down the reaching dynamics. Hence, λ must be selected to balance transient convergence and steady-state smoothness.

Based on the preceding tuning considerations, the final numerical values were selected through a heuristic tuning process while respecting the stability conditions derived from the Lyapunov analysis. The parameters used in this work were set to $K_{\min}=0.3\;\omega_{r,\mathrm{rated}}$, $K_{\max}=4\;\omega_{r,\mathrm{rated}}$, and λ=1. The selected $K_{\min}$ provides adequate local correction near the sliding surface, $K_{\max}$ ensures sufficient convergence capability during speed steps, low-speed operation, and speed reversal, while λ=1 provides an intermediate transition rate between smooth steady-state behavior and fast transient response. Therefore, the adopted parameter set represents a practical compromise between fast convergence, chattering attenuation, and steady-state estimation smoothness for the tested SPIM drive.

### 6.3. Quantitative Performance Assessment

The quantitative performance indices presented in Table 3, Table 4 and Table 5 support the qualitative observations discussed above. Under no-load conditions at 150 r/min, the SMO + ERL achieves lower RMSE values in all stator current components compared with the classical SMO. The improvement is particularly evident in the (x–y) subspace, where the RMSE of $i_{xs}$ decreases from 1.1045A to 0.5790A, and the RMSE of $i_{ys}$ decreases from 1.9396A to 1.0105A. In addition, the MVE of the rotor speed decreases from 2.5927% to 2.0127%, confirming that the proposed ERL formulation improves the speed-estimation behavior and reduces the oscillatory effects observed in the classical LPF-based SMO.

Under load conditions, the proposed observer maintains stable performance and competitive estimation accuracy despite the mechanical load applied to the drive. In this condition, the SMO + ERL reduces the RMSE of the α current component and provides a slightly lower RMSE in the *x* component. However, the β and *y* current components, as well as the speed MVE, present slightly higher values than those obtained with the classical SMO. This result indicates that the adaptive correction introduced by the ERL improves the transient and low-speed behavior but may modify the interaction between the observer dynamics and the current-control loops under loaded steady-state operation. Nevertheless, the overall response remains stable and well-regulated, confirming the robustness of the proposed observer under loaded conditions.

The comparison between operation at 150 r/min and 300 r/min illustrates the adaptability of the proposed observer over different speed ranges. As speed increases, the RMSE values for the current components increase moderately, particularly for the secondary subspace currents, which is expected due to the higher electrical frequency and the more demanding dynamic conditions. However, the MVE remains practically constant, varying only from 2.0127% to 1.9935%, demonstrating that the proposed SMO + ERL preserves accurate speed estimation across the tested speed range.

Overall, the experimental and quantitative results demonstrate that the incorporation of the exponential reaching law substantially improves the adaptive capability of the observer while simultaneously reducing oscillatory behavior and preserving the robustness characteristics of sliding-mode estimation techniques. Therefore, the proposed SMO + ERL constitutes an effective alternative to conventional LPF-based SMO structures for sensorless control of multiphase induction motor drives, providing enhanced transient response, improved low-speed performance, and stable operation under variable operating conditions.

## 7. Conclusions

This paper presents a speed-sensorless control strategy based on a sliding-mode observer with exponential reaching law (SMO + ERL) for an asymmetrical six-phase induction motor operating under an IRFOC scheme. The proposed observer was evaluated through simulation studies and experimentally validated under transient and steady-state operating conditions. The obtained results demonstrated improved dynamic performance during speed-step variations and speed reversals, with smoother current waveforms and reduced oscillatory behavior in both the (α–β) and (x–y) subspaces compared with the conventional LPF-based SMO. In addition, the proposed approach achieved stable operation and accurate speed estimation under low-speed and loaded operating conditions.

A Lyapunov-based stability analysis demonstrated that appropriate selection of the proposed SMO + ERL parameters guarantees global reaching of the sliding manifold and convergence. The ERL parameters $K_{\min}$, $K_{\max}$, and λ regulate the trade-off between convergence speed and chattering attenuation. Specifically, $K_{\min}$ defines the minimum correction level near the sliding surface, $K_{\max}$ determines the maximum correction capability during large transients, and λ governs the rate at which the adaptive gain increases with the magnitude of the sliding variable. In this manner, the adaptive gain mechanism reduces the effective switching intensity near the sliding surface while maintaining fast convergence during large transients, thereby improving the smoothness of the estimated currents and speed signals without introducing the phase delay associated with LPF-based SMO implementations.

The quantitative performance indices confirmed the advantages of the proposed ERL formulation, with reduced current RMSE and improved speed-estimation accuracy across a wide operating range. Overall, the proposed SMO + ERL constitutes an effective alternative for sensorless control of multiphase induction motor drives, combining fast convergence dynamics, reduced filtering-induced delay, improved steady-state smoothness, and stable operation under variable operating conditions. Therefore, the proposed observer provides a computationally efficient solution for real-time sensorless IRFOC applications involving asymmetrical six-phase induction machines.

Future work will focus on the optimization of the ERL gain parameters in order to further improve the trade-off between convergence speed and chattering attenuation, as well as on the integration of the proposed observer with more advanced current control schemes for high-performance multiphase drive applications. In addition, higher-order sliding-mode strategies, such as super-twisting-based approaches, will be investigated to improve the steady-state quality of the estimation signals and mitigate residual chattering effects.

## Figures and Tables

**Table 1:** Parameters used in the comparative tests.

Parameter	Classical SMO [24]	Proposed SMO + ERL
Switching function	$\mathrm{sgn}(S_{bn})$	$\mathrm{sgn}(S_{bn})$
Observer gain	$K_{s}=2000$	Adaptive gain $K_{S_{bn}}$
Minimum gain	–	$K_{\min}=0.3\;\omega_{r,\mathrm{rated}}$
Maximum gain	–	$K_{\max}=4\;\omega_{r,\mathrm{rated}}$
ERL curvature parameter	–	λ=1
LPF type	First-order LPF	Not used
LPF cutoff frequency	$f_{c,\mathrm{LPF}}=0.8$ [Hz]	–
Sampling frequency	$f_{s}=10$ [kHz]	$f_{s}=10$ [kHz]
Discretization method	Euler method	Euler method
Magnetizing current reference	$i_{ds}^{*}=2.5$ [A]	$i_{ds}^{*}=2.5$ [A]
DC-link voltage	$V_{dc}=325$ [V]	$V_{dc}=325$ [V]

**Table 2:** Electrical and mechanical parameters of the SPIM.

Parameter	Description	Value
$P_{n,\mathrm{rated}}$	Rated power of the SPIM	15 [kW]
$\omega_{m,\mathrm{rated}}$	Rated rotor speed	970 [r/min]
$T_{em,\mathrm{rated}}$	Rated torque	140 [Nm]
$I_{s,\mathrm{rms}}$	Rated current	15 [A]
$V_{s,\mathrm{rms}}$	Rated voltage	380 [V]
$f_{e,\mathrm{rated}}$	Rated electrical frequency	50 [Hz]
*P*	Pole pairs	3
*B*	Friction coefficient	0.012 [kg·m^2^/s]
*J*	Moment of inertia	0.27 [kg·m^2^]
$R_{s}$	Stator resistance	0.62 [Ω]
$R_{r}$	Rotor resistance	0.63 [Ω]
$L_{m}$	Magnetizing inductance	0.0666 [H]
$L_{lr}$	Rotor leakage inductance	0.0035 [H]
$L_{ls}$	Stator leakage inductance	0.0064 [H]

**Table 3:** Performance comparison between the SMO and the proposed SMO + ERL at a reference speed of 150 r/min under no-load conditions, considering the RMSE [A] of the stator αβxy currents and the MVE [%] of the rotor speed.

Test Condition: No-Load Operation		
Figure of Merit	SMO	Proposed SMO + ERL
RMSE($i_{\alpha s}$)	3.7168	3.0312
RMSE($i_{\beta s}$)	3.4020	2.7013
RMSE($i_{xs}$)	1.1045	0.5790
RMSE($i_{ys}$)	1.9396	1.0105
MVE($\omega_{m}$)	2.5927	2.0127

**Table 4:** Performance comparison between the SMO and the proposed SMO + ERL at a reference speed of 150 r/min under a load torque of approximately 40 Nm, considering the RMSE [A] of the stator αβxy currents and the MVE [%] of the rotor speed.

Test Condition: Load Operation		
Figure of Merit	SMO	Proposed SMO + ERL
RMSE($i_{\alpha s}$)	3.7773	2.7838
RMSE($i_{\beta s}$)	3.1626	3.5205
RMSE($i_{xs}$)	1.3391	1.3014
RMSE($i_{ys}$)	1.1720	1.4713
MVE($\omega_{m}$)	0.5785	1.5785

**Table 5:** Performance metrics of the proposed SMO + ERL at reference speeds of 150 r/min and 300 r/min: RMSE [A] of the stator αβxy currents and MVE [%] of the rotor speed.

Figure of Merit	SMO + ERL 150 r/min	SMO + ERL 300 r/min
RMSE($i_{\alpha s}$)	3.0312	3.5313
RMSE($i_{\beta s}$)	2.7013	2.7657
RMSE($i_{xs}$)	0.5790	1.1993
RMSE($i_{ys}$)	1.0105	1.1374
MVE(${\hat{\omega }}_{m}$)	2.0127	1.9935

## Data Availability

The contributions presented in this study are included in the article; further inquiries can be directed to the corresponding author.

## Abbreviations

The following abbreviations are used in this manuscript:

CB-PWM	Carrier-Based Pulse Width Modulation
DSP	Digital Signal Processor
FOC	Field-Oriented Control
FPS-PLL	Finite Position Set Phase-Locked Loop
IRFOC	Indirect Rotor Field Oriented Control
EKF	Extended Kalman Filter
ERL	Exponential Reaching Law
RMSE	Root Mean Square Error
MRAS	Model Reference Adaptive System
MVE	Mean Value Error
PCC	Predictive Current Control
PI	Proportional-Integral
SMO	Sliding Mode Observer
SPIM	Six-Phase Induction Machine
VSD	Vector Space Decomposition
VSI	Voltage Source Inverter
